# Predictive Maintenance and Intelligent Sensors in Smart Factory: Review

**DOI:** 10.3390/s21041470

**Published:** 2021-02-20

**Authors:** Martin Pech, Jaroslav Vrchota, Jiří Bednář

**Affiliations:** Department of Management, Faculty of Economics, University of South Bohemia in Ceske Budejovice, Studentska 13, 370 05 Ceske Budejovice, Czech Republic; mpechac@ef.jcu.cz (M.P.); bednaj10@ef.jcu.cz (J.B.)

**Keywords:** intelligent sensors, maintenance, smart factory, Industry 4.0

## Abstract

With the arrival of new technologies in modern smart factories, automated predictive maintenance is also related to production robotisation. Intelligent sensors make it possible to obtain an ever-increasing amount of data, which must be analysed efficiently and effectively to support increasingly complex systems’ decision-making and management. The paper aims to review the current literature concerning predictive maintenance and intelligent sensors in smart factories. We focused on contemporary trends to provide an overview of future research challenges and classification. The paper used burst analysis, systematic review methodology, co-occurrence analysis of keywords, and cluster analysis. The results show the increasing number of papers related to key researched concepts. The importance of predictive maintenance is growing over time in relation to Industry 4.0 technologies. We proposed Smart and Intelligent Predictive Maintenance (SIPM) based on the full-text analysis of relevant papers. The paper’s main contribution is the summary and overview of current trends in intelligent sensors used for predictive maintenance in smart factories.

## 1. Introduction

Industry sets the direction for the world economy, accounting for more than 70% of the world’s total material production [1], depending on the regional economy’s development. The continuous introduction of new technologies in developed countries has an impact on its relatively low level of employment of the population at the level of 20% [2]. Simultaneously, the share of products and semifinished products in international trade is continuously growing, despite the declining share of national Gross Domestic Product (GDP) in developed countries [3]. All these facts are caused by introducing new technologies, which in the current industrial era of Industry 4.0 are summarised by many authors under the name Smart Factory.

This review aims to give the reader a comprehensive view of maintenance and intelligent sensors in Smart Factory. As can be seen from the following, current literature reviews [4,5,6,7,8,9,10] have shown that the literature is focusing on specific topics only separately. The literature specializes in different types of sensors but does not consider them in relation to those technologies, and industry 4.0. Professional texts lack a summary of literature and texts that would bring the usability and potential of sensors closer to common practice, so that these findings can be clearly used for business management in the implementation of maintenance system planning. This would be beneficial for operational managers and engineers for the design of new maintenance systems. This article provides a comprehensive overview of current trends to help structure and guide future research. At the same time, it answers key questions related to contemporary trends in maintenance processes in smart factories. We define which Industry 4.0 technologies and intelligent sensors usually provide maintenance in smart factories. Moreover, it helps to find new trends in smart and intelligent predictive maintenance.

**Figure 1:**
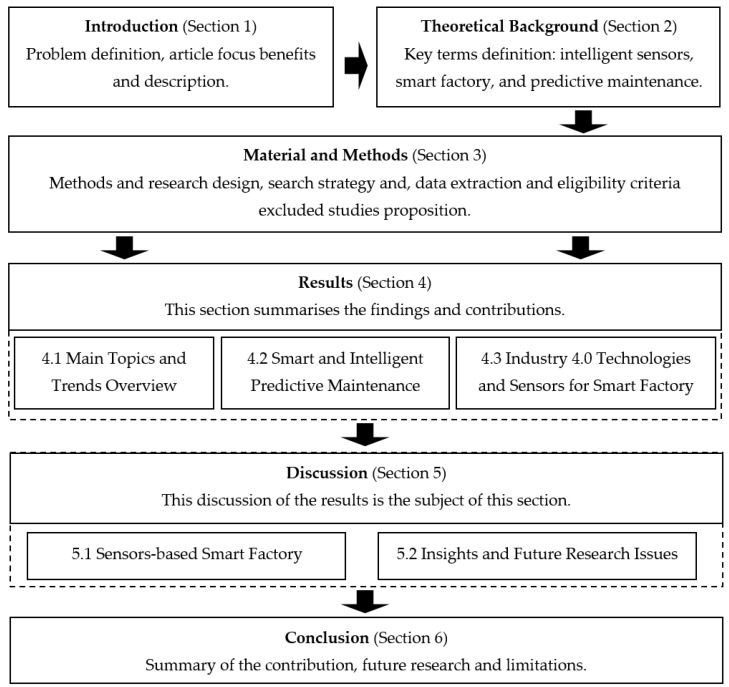
Organisation of the article.

The article is organised into six sections (Figure 1). After the Introduction (Section 1), the Theoretical Background (Section 2) discusses the relevant literature about intelligent sensors, smart factory, and predictive maintenance and defines key terms. In Materials and Methods (Section 3), we explain the qualitative and quantitative methods used for the review. Section 4 is focused on the main results of the article, followed by a discussion (Section 5). The last part presents the conclusion (Section 6), contributions, limitations, and future research.

## 2. Theoretical Background

A literature review discussing intelligent sensors for maintenance in smart factories has not been carried out. The literature currently offers reviews dealing with these areas separately. Song at al. [4] pays attention to smart sensors in monitoring the condition and integrity of rock bolts concerning economic and personnel losses. In the field of engineering, Jin [5] describes multifunctional sensors suitable for industrial production, Feng [11] describes sensors for intelligent gas sensing in the literature review, and Paidi [6] describes intelligent parking sensors replacing ultrasonic sensors in combination with machine learning. Sony and Talal [7,12] then characterise sensors for health monitoring. In literature reviews, we relatively often find a combination of smart sensors and smart factories, a key part of the 4.0 industry concept [13]. Lee [9] describes smart sensors’ use to evaluate and diagnose individual devices in a smart factory. Strozzi [10] expands the literature review emphasising the actual transition and implementation of large, intelligent factories. Pereira and Álvarez [14,15] also focus on implementing the principles of a smart factory and emphasise that effective value creation depends on the method of implementation. The implementation process about managing technological and organisational changes and desirable competencies is further addressed by Sousa [16], as well as Lee et al. [17]. They pay attention to the gap between recent researches on the actual level of deployment. In the field of maintenance of intelligent factories, we find several literature reviews with the resonant notion of predictive maintenance. In their overview, Carvalho [18] focuses on machine learning methods, which they consider a promising tool for predictive maintenance. Sakib [19] observes the shift from service activities to proactive, predictive maintenance and places [20] in the context of Industry 4.0. Olesen and Shaker [21] deals with practical use in thermal power plants, and Fei [22] in the field of aircraft systems.

### 2.1. Intelligent Sensors

We find a critical area in the concept of smart factories, and that is intelligent logistics—transport and warehousing. These operations include identification and detection of the location of resources across the company. We understand sensors as a technical reproduction of natural processes because human nerves transform external stimuli into electrical signals transmitted to the central nervous system—the brain. Smart sensors process aggregated data from production processes in real-time and enable self-determination of machines and other smart devices [23,24]. Data on production resources are used to monitor, collect, and evaluate the data obtained. The most common type of wireless sensors work on radio frequency identification (RFID), ZigBee, and Bluetooth technology [25]. Eifert [26] defines intelligent sensors as a multi-component measuring device that is self-calibrating, self-optimised, and easy to integrate into the environment for high connectivity. Besides, intelligent sensors also have process intelligence and can generate multidimensional data information. The literature of the most inflected RFID technology is also one of the key technologies for implementing the Internet of Things (IoT). In RFID technology, it is an active and passive tag, where the active sensor sends information for hundreds of meters, and the passive tag receives it. The integrated sensor in production enables a flexible and targeted strategy of predictive maintenance and control [27]. Sensors that are part of the IoT can proactively monitor the device and issue alerts when the device deviates from the specified parameters, we speak of so-called facility management [28]. Karabegovic [29] adds that the sensors convert physical parameters (temperature, speed, humidity) into signals that can be measured electronically. The use of smart sensors in Industry 4.0 is characterised by Schmitt [30]. The key element is knowledge of the current state of the system. Sensor data with other parameters of the given process in the sense of self-diagnostics and machine learning can be evaluated in real-time. The traceability of individual components is also essential—from screws to the uniformity of seals, this knowledge enables the measurement of tolerance and comprehensive monitoring of the condition of machines and other equipment.

### 2.2. Predictive Maintenance

**Figure 2:**
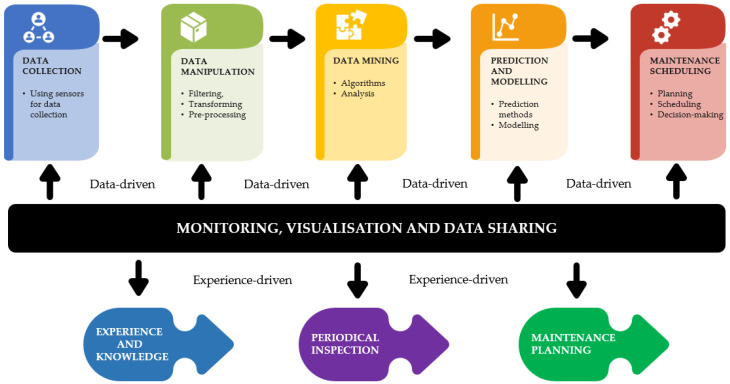
Experience- and data-driven predictive maintenance [38].

Predictive maintenance (PdM) is the latest maintenance policy adopted by many industries. Above all, these are areas that require absolute reliability, such as power plants, public services, transport systems, and emergency services. Forecasted information is usually necessary for the long term and for planning various operational activities (maintenance, production, inventory, etc.). In addition, due to technological and logistical limitations, maintenance cannot always be performed everywhere [31]. Maintenance is a critical activity that takes place in production. Machine failures during production can lead to adverse effects on the production schedule, delivery delays, or employee overtime to compensate for the loss. PdM predicts system failures to optimize maintenance efforts [32,33]. According to Carvalho [18,34], PdM is a set of tools used to determine when specific maintenance is required. The tool is based on continuous monitoring of the machine or process, and this allows maintenance to be performed only when needed. A secondary, no less important function of PdM is the possibility of early detection of faults, thanks to tools based on historical data—machine learning—as well as visual aspects of faults—colour and wear. As a possible part of the Industry 4.0 concept, PdM aims to minimise maintenance costs, implement zero-waste production, and reduce the number of major failures [35]. Despite PdM’s benefits, Herrmann [36] highlights the potential risks of remote access to maintenance processes and cites Distributed Denial-of-Service (DDoS) attacks, for example. According to Zonta [20], we distinguish three approaches to PdM, namely: Based on a physical model, where the main feature is mathematical modelling requiring the timeliness of the state and statistical methods of evaluation. The second approach is the knowledge-based approach, which reduces the complexity of the physical model, and the last approach is the data-driven approach, which we find most often in the current development of PdM. This approach is based on artificial intelligence, i.e., machine learning and statistical modelling, and is a satisfactory approach in the conditions of Industry 4.0 [37]. Farooq et al. distinguish experience-driven and data-driven maintenance [38]. Experience-driven preventive maintenance is based on gathering knowledge about production equipment, which is then used to plan future maintenance. On the contrary, data-driven preventive maintenance is based on analysing a large volume of data (Figure 2).

### 2.3. Smart Factory

One of the key components of Industry 4.0 is a smart factory, otherwise also a smart or digital factory. A smart factory represents the future state of fully interconnected production systems, without a significant amount of manpower [13]. According to Chen [25], a smart factory is a manufacturing solution that provides adaptive and flexible manufacturing processes that reflect the rapidly and dynamically changing conditions in the world. On the one hand, this solution can be understood as a combination of software and hardware, which should lead to the optimisation of production and the reduction of waste of scarce resources. On the other hand, the concept can be perceived as a space for perspective cooperation with business partners based on the formation of a dynamic organisation. The smart factory architecture itself includes a physical resource layer, a data and network layer, as well as an end layer. All elements of the intelligent factory are interconnected, exchanging information, and recognising and evaluating situations. Thus, physical and cyber technology is integrated, which results in improved controllability, control, transparency of production processes, maximises value for the customer, and in addition, there is communication between the factory and the market itself [39,40]. The core technologies of Industry 4.0 include IoT, cloud computing, and high-volume data analysis. IoT represents the integration of sensors and computer technology in the field of wireless communication, and cloud services allow access to the network respectively, as a shared pool of computing resources. The combination of these technologies allows the involvement of all devices in the concept of a smart factory, but the collection of huge amounts of data requires another technology, which is the analysis of high-volume data. With the help of analytical tools—data mining or machine learning—this technology is one of the most important elements of the entire concept of Industry 4.0 [41]. Other technologies related to the vision of a smart factory include autonomous robots, additive manufacturing, augmented reality, and cybersecurity. Drastic changes in technology are changing the concept of production. In the modern concept, traditional centrally controlled processes are replaced by decentralised control, which builds on the ability of individual elements of a smart factory to communicate with each other. In self-regulatory production, people, machines, equipment, and products communicate with each other [42].

## 3. Materials and Methods

The paper is based on a review of the literature focusing on intelligent sensors, maintenance, and smart factories. As part of this review, we analysed, evaluated, and discussed scientific publications from prestigious databases. This review focuses on combining three concepts and creating a comprehensive overview of studies to find possible research gaps. The literature review has shown that the current literature contains many reviews focusing on particular topics only separately. This paper provides an overview of the contemporary trends that will help structure and guide future research to fill this gap.

The scope of the paper is to review intelligent sensors and maintenance processes in smart factories. As part of the research, we formulated the following research questions:

1. What are contemporary trends in maintenance processes in smart factories?
2. How are Smart and Intelligent Predictive Maintenance characterised?
3. Which Industry 4.0 technologies and intelligent sensors provide maintenance in smart factories?

### 3.1. Research Design

**Figure 3:**
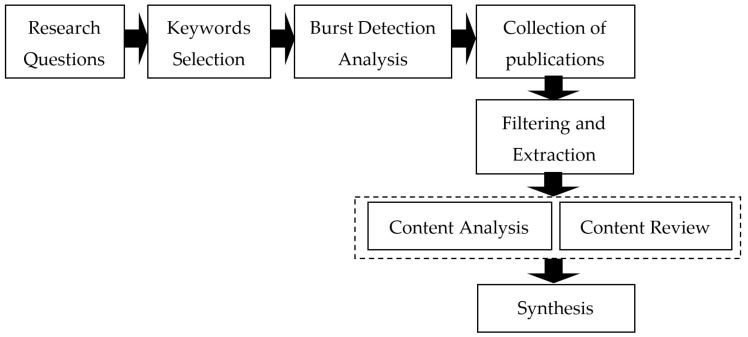
Research design and procedure.

The research procedure and strategy phases are described in Figure 3. First, we defined research questions that helped with keyword selection. Then, we analysed these keywords separately through burst detection analysis to reveal current research trends. Data was collected from database sources, filtered, and extracted based on predefined criteria. Further, we performed a content analysis and review of individual publications in our research team. Based on the analyses, we presented results of the synthesis.

### 3.2. Keywords Selection

We used the modified Eligibility methodology [43] to prepare the research questions and achieve the review’s scientific quality. The four parts of this methodology (Problem/context, Intervention, Comparison, Outcomes) create the PICO logic grid [44]. We do not use the comparative part, and we have expanded the method into three main questions. The question “how” is related to the context smart factory. The question “which” refers to interventions via sensors that are drivers for bringing a change from the traditional concept of maintenance. Finally, the question “what” focus on outcomes for maintenance. We used software VOSviewer [45] for constructing keywords in the PICO logic grid (Table 1).

### 3.3. Burst Detection Analysis

We examined research topics (smart factory, intelligent sensors, predictive maintenance) separately in this phase. We collected the required data from the Web of Science (WoS) and Scopus databases. Furthermore, we analysed the obtained publications concerning their trend over time, keywords, and burst detection. Only the most relevant research publications from the WoS database are analysed. The search strategy is shown in Table 2.

Burst detection analysis is based on the analysis of keywords in a certain period of time [46]. Density of the frequency changes of keywords are determined for each monitored period. This analysis helps to identify the main research trends and helps to predict the future evolution of the literature. Burst analysis can be performed in the Sci2 Tool [47], which offers a display of the results of temporal bar graph promotions. The number of publications for burst analysis are presented in Table 3.

### 3.4. Collection of Publications for Review

The systematic search strategy (Table 4) combines various keywords and their synonyms to find out quality peer-reviewed journals. In this phase, we searched the publication’s databases Web of Science and Scopus to combine all three main terms. Our goal was to obtain publications that connect topics, smart factories, intelligent sensors, and maintenance.

We present the total number of publications found in Table 5. The result of the search was 890 publications, which we further filtered based on the selected criteria.

### 3.5. Data Extraction and Eligibility Criteria

Data extraction is based on the filtering criteria determining the selected publications in more in-depth research. We filtered search results based on the criteria:

1. Not duplicated,
2. Published from January 2010 to December 2020,
3. Written in English,
4. Type of publication: journal paper (not review, white paper, book, short survey, proceedings, conference paper, etc.) for higher quality of data,
5. Publications with completed information (authors, year, journal name, etc.).

**Figure 4:**
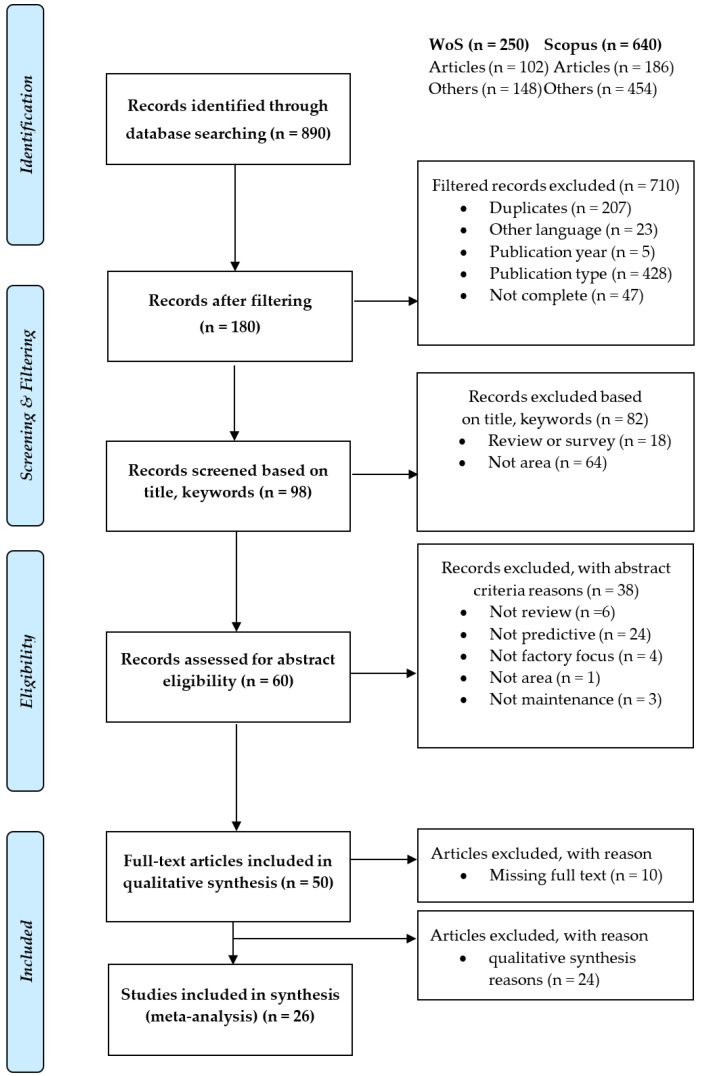
Flow diagram based on PRISMA [48] and QUORUM [49] flowchart.

Furthermore, an objective screening was performed based on the title and keywords. To evaluate the eligibility, we analysed the title, keywords, and abstracts of publications. For this purpose, we have defined criteria for exclusion. We evaluated the publications at meetings of the research team. In case of discrepancies in the assessment of suitability in the title, keywords, or abstract, we compared the opinions of team members and, if necessary, performed a full-text analysis. To document the extraction process, we used the flow diagram in Figure 4, which captures the entire review flow based on the Preferred Reporting Items for Systematic Reviews and Meta-Analyses (PRISMA) methodology [48] and Quality of Reporting of Meta-Analyses (QUORUM) methodology [49] from search to final selection. The PRISMA checklist is available in supplementary materials (Table S1).

The exclusion criteria are:

1. The paper is a review (paper is focused only on challenges or future perspectives).
2. The paper focused on maintenance, but not on “predictive” maintenance.
3. The paper does not discuss Industry 4.0 technologies.
4. The paper does not have a production/factory focus (for example, we eliminated papers related to smart cities and smart homes, agriculture, logistics, etc.).
5. The paper has unavailable full text.
6. The paper does not have a scientific structure (abstract, introduction, literature background, methodology, results, discussion, conclusion, references).

### 3.6. Content Analysis and Synthesis

The results of search query publications were further subject to analysis and synthesis. We performed the analysis in the Endnote software database and Microsoft Excel software, which were used to evaluate various aspects of the monitored topics. Our research team consisted of three members who participated in the evaluation and analysis of search results. This phase included evaluating the full texts of individual publications based on the set research goals.

We focused mainly on:

- The research method (case study, experiment, simulation)
- Objectives of the articles
- Used intelligent sensors and data
- Industry 4.0 technologies for smart factories
- Predictive maintenance processes characteristics
- Smart and intelligent keywords domain

We used the clustering method during the analysis, which is part of the VOSviewer software [45] and allows classification using keywords. Using VOSviewer, we performed a co-occurrence analysis of keywords, which is based on clustering methods. Cluster analysis enabled to find a number of paired keywords cited in the same publications. The results of the cluster analysis clearly capture the knowledge structure of the research frontiers. We evaluated the quality of publications included for the synthesis (final selection) thorough review of individual team members. In particular, the ranking of journals, citations of publications, and their possible biases were considered.

### 3.7. Excluded Studies

To avoid possible effects of bias, it is necessary to mention the most important publications, which were eliminated from the sample for qualitative synthesis reasons. Aheleroff et al. [50] examined the application of smart IoT sensors in the fridge (smart homes focus). Several publications are mainly focused on the logistic area [51], i.e., from the automotive industry [52,53,54] or the aviation industry [55,56]. We excluded from the analysis publications focused on agriculture, which do not deal with smart factories’ production processes. These are publications using intelligent sensors in milling [57] and aquaponics [58]. The review papers were eliminated from the final selection.

## 4. Results

The results are divided into three parts according to the research question: an overview of the main topics, smart and intelligent predictive maintenance, and Industry 4.0 Technologies and Sensors for Smart Factory.

### 4.1. Main Topics and Trends Overview

First, we identified occurrences of the primary topics “smart factory/production,” “intelligent/smart sensors,” and “predictive/smart maintenance” in the Web of Science and Scopus databases. This part of the research related to the research question 1. All three concepts are connected in the research area by engineering, supplemented by telecommunications and predictive maintenance by medicine, or intelligent sensors in computer science. The total number of publications in the databases is shown in Figure 4.

**Figure 5:**
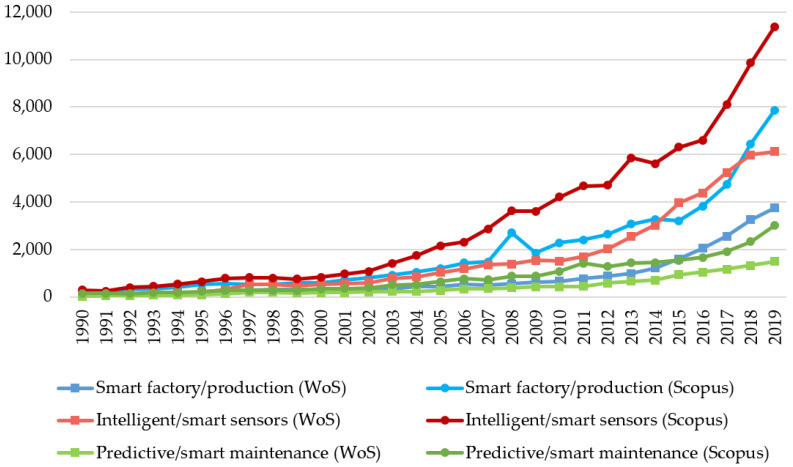
The development of the total number of Scopus and Web of Science publications. Note: square on the line (Web of Science), circle on the bold line (Scopus).

The results shown in Figure 5 show an increase in the number of publications over time. This increase has been apparent for smart factory publications since 2015 and intelligent sensors since 2012. Further, we performed burst detection analysis in the researched areas for Web of Science publications.

**Figure 6:**
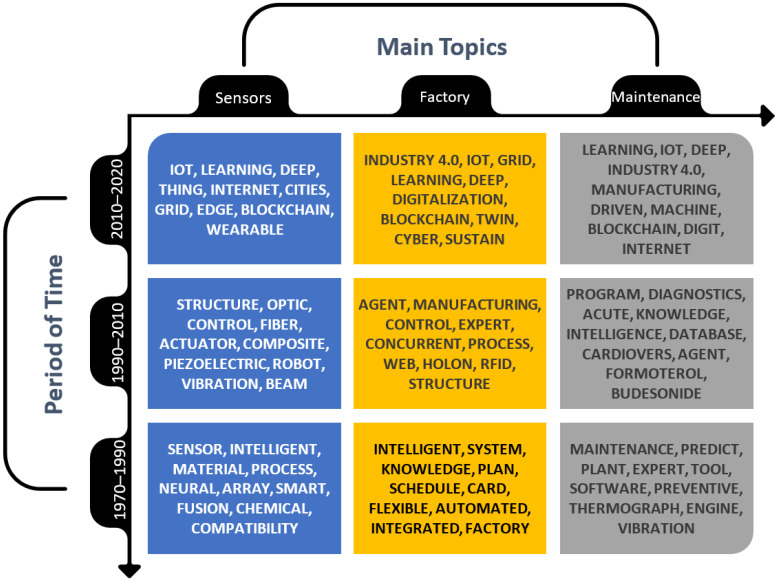
The summary of burst detection analysis for main topics. The results are divided into three periods of time (1970–1990, 1990–2010, and 2010–2020). The terms in each period are sorted according to the burst weights.

**Figure 7:**
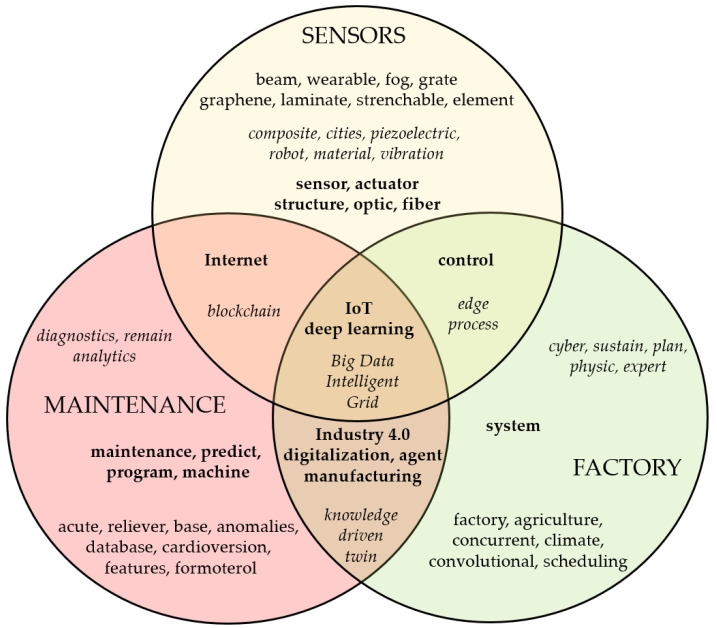
The top terms in analysed areas based on the burst detection. Note: The top ten used terms are highlighted “bold” and top twenty terms are depicted “italic”.

The burst detection analysis presents key terms for topics of intelligent sensors, smart factory, and predictive maintenance (see Figures S1–S3 in Supplementary Materials). For a better overview, we compare the results in three time periods (Figure 6), and then according to the individual importance of key concepts (Figure 7). The importance of the terms was expressed using the obtained burst weights. Based on these findings and analysis, we tried to answer research question 1 responsibly.

Top cited papers from smart factory/production areas focus on using ion batteries for smart grids [59,60] and nanomaterials’ intelligent design [61]. The results show that the most used terms in the paper titles are intelligent, Industry 4.0, and agent. Based on Figure 5, we conclude that the oldest wave in smart factories is associated with classical studies dealing with intelligent, flexible, and automation planning and scheduling of manufacturing systems. This wave is the period 1970–1990, characterised by the burst terms intelligent, system, knowledge, plan, and schedule. The second wave in 1990–2010 with the primary burst terms: agent, manufacturing, control, expert, and process, refers to papers using holon, RFID, or web technologies in factories. Publications on manufacturing control systems [62,63] were highly cited in this period. The current trend in smart factory is related to implementing intelligent manufacturing [64]. In this contemporary wave, burst terms Industry 4.0, digital twin, IoT, deep learning, digitalisation, smart grid, cyber, and sustain dominate. These terms are well-known Industry 4.0 technologies and processes. Top cited papers focused on operational planning of a smart grid [65], deep learning in agriculture [66], and big data for the self-organised multiagent system in the smart factory [67].

Top cited papers from smart/intelligent sensors were in areas related to the Internet of Things [68], wireless sensor networks [69], and nanotechnology applications [70]. We found that the important paper title terms are IoT, structure, and sensor in the burst analysis. Figure 5 shows that the early history of intelligent sensors, 1970–1990, emphasised the first application of sensors (burst terms sensor, intelligent, process). Later, in 1990–2010 came articles focused on the structure [71], optic, and control of sensors, and their usage for robots. Some essential publications in this period focused on structural health monitoring [72], piezoelectric laminate beam [73], and free vibration behaviour of the beam [74]. The most contemporary period from 2010 to 2020, similar to the smart factory/production, covered the area of Industry 4.0 new technologies. In addition to the mentioned Internet of Things [75], there is a significant representation of publications focused on wearable sensor-based systems [76], deep learning [77], edge technology [78], graphene-based smart materials, blockchain, smart city, and grid.

The last area focused on smart/predictive maintenance. After omitting medical and ecological articles, the most cited publications focused on proportional-integral-derivative (PID) control [79], monitoring, and fault diagnosis in production [80]. Based on the burst analysis results, we found that the most important terms are maintenance, learning, and predict. We identified three trend waves in area maintenance (Figure 5). In the first wave from 1970 to 1990, the publications dealt with predictive maintenance. In engineering and production, maintenance is associated with predicting machines’ status [81] or deterioration of processes [82]. In the second wave in 1990 to 2010, we found that the publications dealt with burst terms program, diagnostics, intelligence, knowledge, and database. These publications focus, for example, on diagnostics, monitoring, or maintenance of intelligent computer numerical control (CNC) machine tools [83] or power transformers [84]. The current trend wave is characterised by Industry 4.0 technologies such as digital twins, deep machine learning, IoT, big data analytics, blockchain, and digitisation for maintenance. The most significant publications of this period focused on big data analytics in logistics and supply chain management [85], maintenance strategy selection [86], vibration analysis of rotating machinery, or cloud-enabled prognosis [87] for predictive maintenance in production.

Based on the burst analysis detection, we conclude that in all three areas in the last 10 years, the focus has been on the concept of Industry 4.0 and related technologies. We arranged the keywords with the highest burst weights into three research areas in Figure 7. The results show that the terms Internet of Things and deep learning have the highest weight for all topics. The terms Big Data, grid, and intelligent are also common to the area. From this finding, we can conclude that the current trend in the monitored areas is related to the collecting of big data through intelligent sensors on IoT devices and their evaluation using learning algorithms.

The internet, smart grid, and blockchain technology are important for sensors used in maintenance. The use of sensors in smart factories lies mainly in the area of control, with a focus on processes. The sensors, together with actuators, are used to collect data to control and optimise conditions. Piezoelectric, optics, wearable, beam, graphene, and other sensors’ features are used. A special area of sensors lies in robotics, which has experienced rapid development in recent years. In the world’s most industrialised countries, such as South Korea, Japan, Germany, and Sweden, there is the largest share of robots per 10,000 employees in factories [88]. Automation in smart factories requires new types of sensors that have the ability to automatically calibrate and improve the functions of IoT devices. The IoT is not aiming only at connect two machines with pre-programmed functions. For IoT communication, it is important to connect embedded devices to the Internet and communicate with each other [89]. It is an intelligent connection of various products, devices, and facilities that provide a wide range of functions that evaluate certain conditions. The interaction between systems brings new possibilities. The key elements are miniature intelligent sensors [90]. Even though devices and systems were not originally designed to share data, the Internet of Things can. Connecting smart sensors and gateways to existing devices leads to data collection and analysis, understanding, and better decision making [91].

Publications about smart factory/production are related to cyber-physical systems, planning, scheduling, and sustaining them. Maintenance in smart factories relies on Industry 4.0 technologies, digitisation, data-driven manufacturing, agent-based systems, and digital twins. Predictive maintenance consists of programs for predicting, diagnosing, and analysing future maintenance needs. Based on the rules, features, and conditions, there are machines and devices controlled and repaired to maintain their life and future sustainability. Information and data are collected and shared through databases.

### 4.2. Smart and Intelligent Predictive Maintenance

**Figure 8:**
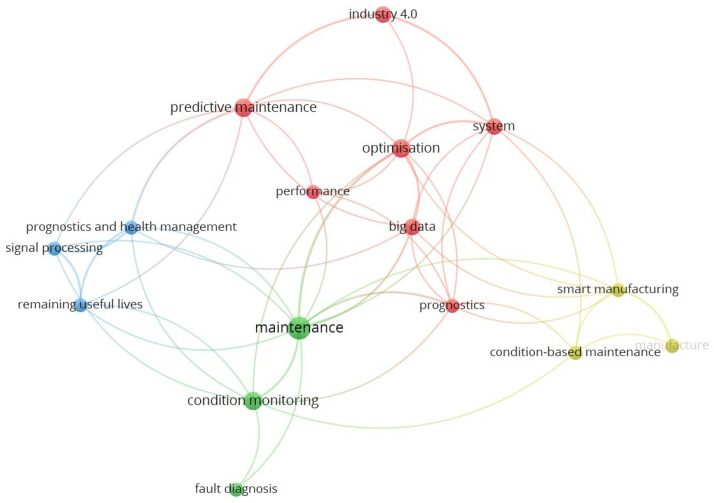
Keywords of co-occurrence analysis.

We performed a co-occurrence analysis based on original papers’ keywords using VOSviewer. This part of the analysis related to research question 2. The results show that the keywords maintenance, optimisation, predictive maintenance, system, and big data were most often used in publications. Publications were grouped based on keywords into four clusters (Figure 8).

- I4: Industry 4.0 for predictive maintenance in general (keywords: Industry 4.0, Big Data, prognostics, optimisation, performance, predictive maintenance, system).
- CbM: Smart manufacturing for condition-based maintenance (keywords: smart manufacturing, manufacture, condition-based maintenance).
- SFD: Condition, state, and fault diagnosis for maintenance (keywords: maintenance, condition monitoring, fault diagnosis).
- RUL: Prognostics and health management for RUL (keywords: prognostics and health management, signal processing, remaining useful lives).

The first cluster consists of publications that focus on intelligent sensors in smart maintenance factories without preferring specific methods. This cluster is represented, for example, by publications focused on data-driven simulation [92], big data in an Industry 4.0 environment [93], or performing predictive maintenance in a bottling plant [94]. The second cluster consists mainly of publications that emphasise the use of condition-based maintenance. The intelligent condition-based maintenance uses data fusion [95] and the Internet of Things in connection to learning techniques [96]. The third cluster related to publications mainly emphasised fault diagnosis’ importance for monitoring and maintenance. The fault diagnosis is used for prognosis in signal processing [97] and maintenance management systems [98]. The last cluster is characterised by a focus on determining the current health and the remaining life of devices and machines. This concept is described concerning edge-cloud platforms [99].

While the concept of condition monitoring has been around for some time, the market for more sophisticated predictive maintenance products is still very young. There are four types of maintenance classified in the literature: corrective, scheduled, condition-based, and statistical-based maintenance [100,101]. Predictive maintenance has evolved from corrective maintenance using new technologies and procedures for predicting and preventing failure. Corrective maintenance is based on the reactive strategy to the maintenance process—however, with a proactive strategy related to the preventive or opportunistic approach. Preventive maintenance is then seen as condition-based, dynamic predictive, or scheduled (periodic) maintenance. The corrective maintenance is based on the repair or replacement of assets ex-post. Condition-based maintenance means the decision-making process, usually in real-time, based on selected indicators computed from the gathered data.

Table 6 depicts maintenance process characteristics from analysed papers. The condition-based preventive maintenance is discussed in Farooq et al. [38], Kumar et al. [102], Li et al. [96], Lin et al. [103], Musselman and Djurdjanovic [104], Yan et al. [93], and Sadiki et al. [105]. Preventive maintenance is regular maintenance of machines, devices, and equipment to prevent their downtime concerning failure state. The preventive maintenance actions were classified by Doostparast et al. [106] as inspection, low-level repair, and replacement. These actions are based on fault prediction time statistically, upon failure accident, time-based (at the age for old machines), or cycle-based (periodically).

### 4.3. Industry 4.0 Technologies and Sensors for Smart Factory

**Figure 9:**
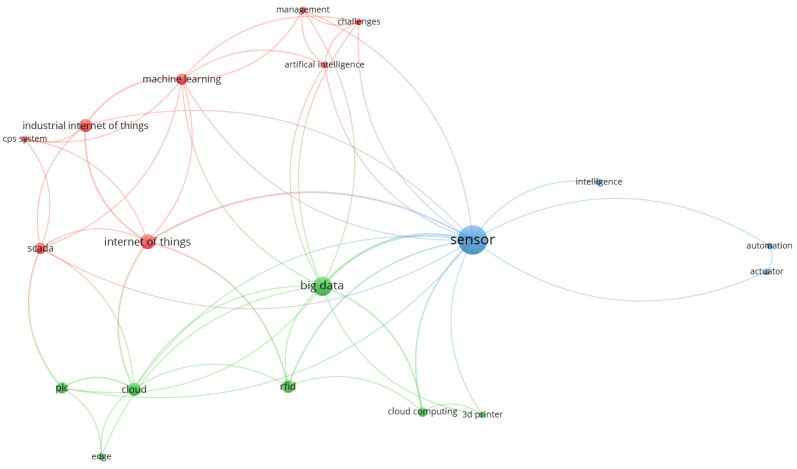
Analysis of keywords related to Industry 4.0 technologies.

Furthermore, full texts of articles concerning Industry 4.0 technologies were analysed. We performed a cluster analysis of the obtained keywords of Industry 4.0 technologies. The results of the analysis are shown in Figure 9. The most common keywords in the articles were sensor, big data, Internet of Things, machine learning, and cloud. Through cluster analysis, we found three clusters:

- A: Intelligent sensors (keywords: sensor, actuator, intelligence, automation).
- B: Cloud-related technologies (keywords: cloud, cloud computing, Big Data, RFID, edge, PLC (programmable logic controller), 3D printer).
- C: Internet of Things technologies (keywords: Internet of Things, SCADA (Supervisory Control and Data Acquisition), CPS (cyber-physical system), machine learning, artificial intelligence, management, challenge).

Table 7 presents the results of classification of researched papers according to belonging to the clusters. The intelligent cluster sensors mainly focused on sensors in general. Kumar et al. [102] analysed remaining useful life (RUL) of cutting machines by a polynomial regression method. Musselman and Djurdjanovic [104] analysed production belt for automation of material handling in the semiconductor industry. The second cluster is focused on Cloud-related technologies. It means that sensors based on RFID [92] and programmable logic controller [107] are used for cloud or edge computing [99] and analysis of big data [109]. The third cluster concerned the IoT technologies based on CPS systems [38], SCADA [94], and data for deep and machine learning.

The different sensors’ data are used for prediction and diagnostics of devices, machines, facilities, and equipment. The results in Table 8 show that data are usually collected from SCADA systems, PLCs, CNC machine sensors, IoT devices, or other special sensors. Analysed papers mostly used case study and experimental research methods.

If we focus closely on individual types of sensors used for predictive maintenance, we will find a number of them, and we can categorize them according to the method of detection of the desired variable. The types and descriptions of sensors used in publications are shown in Table 9. Furthermore, the sensors are elaborated in more details. Vibration and temperature sensors were most often used for predictive maintenance.

#### 4.3.1. Motion, Position, Proximity, and Speed Sensors

The first type of sensors are motion-based probes. Position and movement sensors are mounted for monitoring the machine or product position on the production lines. Inductive, photoelectric, potentiometric (resistance-based), capacitive, optical, magnetic, and other sensing methods are used for detection of position. Sensors based on motion detection must meet the requirements in the areas of durability, weight, energy consumption, and at the same time, suitability for mass production regarding to the end user—customer [123,124]. Shoabid [125] describes motion-based sensors as a combination of an accelerometer, gyroscope, magnetometer, and linear acceleration. The application of these sensors can be found primarily in the field of healthcare systems, with various combinations of the above-mentioned sensors. A gyroscope is used mainly for gait analysis, fall detection, or gesture recognition, or in combination with an accelerometer. Proximity sensors detect the presence of an object without contact. These sensors are based on the optical, ultrasonic, inductive, and capacitive nature. Wearable sensors are able to monitor, for example, physiological parameters in real time.

Speed sensors have an opportunity for detecting object speed (usually for wheels, motor, or rotating particles). Enterprises use speedometers, accelerometers, light detection and ranging (LIDAR) sensors, tachometers, Doppler radars, etc. Farooq et al. [38] discussed genetic-algorithm-based prediction process for intelligent maintenance of textile spinning systems. They used vibration and speed data in a multiagent system for tracking discrepancies and error distribution of machine processing. Integrated Electronics Piezo-Electric accelerometers have been frequently used for machine vibration measurement. Peng et al. [113] use them for an automatic health condition diagnosis without field worker maintenance effort. The results show that dynamic response signals from the accelerometer increased the completeness and performance of the vibration diagnosis function. Further, Peng and Tsan [98] developed a sensor diagnosis and monitoring system to classify the health condition of the online integrated IEPE accelerometer. The solution was integrated into a production line.

Goodall et al. [92] developed, based on the RFID, a data-driven simulation for controlling work-in-process parts in a remanufacturing process and determining the time for operators to process them [104]. Park et al. [112] performed experiments on servo motor lifespan using an accelerated degradation testing method based on thermal stresses. The experimental data are used sensors for monitoring the torque, position, electrical resistance, and moment of inertia of rotor. Shan et al. [114] presented the system architecture and hardware for the welding line, which provides the real-time fine-grained visualisation of the welding robot operation status. The electrocardiogram of intelligent manufacturing equipment technology provides the maintenance of intelligent manufacturing equipment.

#### 4.3.2. Vibration and Torque Sensors

Vibration sensors are used for monitoring the acceleration machine vibration, indicating a potential machine issue. Some sensors have modern fast Fourier transform signal processing to detect failures in machine components. Vibration sensors are the core of preventive maintenance and provide the condition of the device determination.

Barbieri et al. [99] proposed autonomous health management prognostics for smart manufacturing via on-board sensors. Kiangala and Wang [94] integrated a practical use of intelligent sensors in a small bottling plant. Predictive maintenance is used for detecting early faults and failures in conveyor motors. Uhlmann et al. [117] developed a smart sensor network for condition monitoring in factories. Collected data from MEMS sensors are processed in the cloud services and visualised on the mobile platform.

Torsion (torque, rotational) sensors convert a torque reaction and rotary into electrical signal. These sensors measure stationary or dynamic variables, usually in motors, turbines, or generators. Kozlowski et al. [110] used a torque sensor for designing a classifier for cutting tool condition assessment in RUL prediction. Kumar et al. [102] evaluated and estimated RUL for particular failures in distinct health states and faults. Vlasov et al. [119] used wireless vibration sensor networks that allow real-time analysis of the state of the electronic equipment (motor). The purpose of their approach is to minimize the cost of maintenance and develop a system of predictive maintenance for optimisation predictive repair. Zhang et al. [121] used vibration sensors for accurate prediction of the remaining useful life of the rotatory machines. Deep learning model combined a long short-term memory neural network with an attention mechanism for maintenance in mechanical manufacturing.

#### 4.3.3. Acoustical, Sound, and Ultrasonic Sensors

Another group of sensors are sensors focused on sound detection, usually via microphone devices. When a signal is detected by a sound sensor, the level of voltage is translated to the appropriate sound level. Kaptan [126] describes the location of city buses using sound sensors and an accelerometer instead of the standard global positioning system (GPS) location. In such a scenario, the accelerometers detect the movement of the vehicle and the microphone sensing distinguishes the sound level inside and outside the vehicle. Compared to GPS location, energy savings of up to 46% occur. Another possible usage of acoustic sensors is described by Ryu [127] in the field of material detection. Using machine learning techniques, sound sensors are able to estimate relevant information such as the character of an object and its location. Ultrasonic sensors are non-contact devices using the flight of the sound wave greater than that of the human audible range. Similarly, as with sonar, the measurement is based on the Doppler Shift principle. Yan et al. [120] conducted fusion data mining to predict the remaining life of a key component of machining equipment by multisource sensors (acoustical, vibration, optical, or power).

#### 4.3.4. Pressure, Force, Touch, and Tension Sensors

Pressure sensors identify the pressure deviations in the measurement objects or environment. The change detection is usually based on barometric, piezoelectric, capacitive, optical, or resonant sensing principles. Examples of these types of sensors are Bourdon tubes, diaphragms, pressure gauge, or manometers. Tension sensors help with the deformation and movement of the belt automated material handling system monitoring for intelligent condition-based maintenance [104].

Force sensors monitor tensile compressive force signal and translate it into an output electric signal. Their application includes lead cells, strain gauges, or sensing resistors. Very popular are piezoelectric and magnetostrictive technologies. Another way of sensing is based on induction, pneumatic, and hydraulic forces.

#### 4.3.5. Optical, Light, and Machine Vision Sensors

Another type of sensors are sensors with a machine vision function. Machine vision technology has grown significantly in recent years and is becoming part of autonomous vehicles, intelligent systems, and robotics. Optical sensor input into the systems makes these systems intelligent. Visual data are captured in the form of a series of images and after the digitisation process are processed using a machine learning algorithm [128,129]. In the field of material wear detection, three-dimensional (3D) sensors are also used, which represent new technical means for obtaining information. Three-dimensional data provides more information and at the same time, reduces the deviation of the measured data [130]. A large group of sensors consists of chemical character sensors. Advances in chemical, sensing, and wireless technologies have accelerated the development of wireless chemical sensors. These devices allow the collection and distribution of biochemical information. The use of these types of sensors can be found in the areas of environmental or health monitoring [131,132].

Tarashioon et al. [115] described the design of solid-state lighting products based on the reliability system diagnostics (self-maintenance). The light sensor design using light-emitting diode technology is used to monitor system ambient light.

#### 4.3.6. Temperature Sensors

Temperature sensors usually detect changes in machine condition or critical state in the factory (especially in hazardous environments.). The sensors obtain temperature information directly (resistive temperature detectors, thermistors, and thermocouples) or indirectly (infrared sensors). Some of these sensors have a temperature display. Infrared (IR) sensors work on the basis of the optical principle using light. We distinguish reflective and transmissive IR sensors. Reflective IR sensors’ transmitter and detector are positioned adjacent to each other facing the object. Transmissive sensors use LED and photo diodes to detect the object passing between them.

Another group of sensors consists of probes measuring temperature and humidity. Advances in biomaterials offer opportunities to design electronics with unique mechanical stability capabilities, i.e., sensors whose material composition offers the possibility of application in medical implants and disposable wearable devices. Suitable applications can be found in the accurate scanning of biological tissues, internal organs, but also in the textile and food industry [133].

Bekar et al. [108] analysed the real-world industrial data to implement the PdM strategy for the manufacturing enterprise. They evaluated the quality of the process, vibration and temperature data by understanding outliers, and developed maintenance solutions. Sadiki et al. [105] show the advantages of condition-based maintenance for real-time intelligent monitoring for the industrial machine. Tmote sky sensors network and Z1 mote operate through the edge router and enhance the maintenance simulation’s purpose. Villalobos et al. [118] introduced a flexible forecaster analyser system for anticipation of alarms’ activation based on the temperature sensor data. The deep learning techniques based on the short-term memory recurrent neural network contribute to the predictive maintenance approach.

#### 4.3.7. Liquid, Flow, Gas, and Chemical Sensors

Flow sensors enable the possibility to analyse the cooling water and lubrication flow rate. These sensors use magnetic, ultrasonic, or thermal detectors to monitor the current intensity in the pipeline. Chien and Chen [109] used mass flow controllers for monitoring mode and position of silane reactant flow. Their research is related to a data-driven framework for monitoring equipment’s health condition (RUL).

Oil particle sensors enable the possibility of monitoring contamination levels in lubrication systems (for example, gear boxes). These sensors target to change the level of pollution based on the presence of the number of substances processed. They analysed the light intensity via a laser beam and photo detector.

Humidity (moisture) sensors focused diagnostics on water content in oils to prevent corrosion of machines. These sensors are usually installed in a lubrication or hydraulic tank. Humidity sensors play an essential role in the selected automated manufacturing processes. To achieve the desired atmosphere, it is necessary to detect, monitor, and regulate humidity in conditions of low and high temperatures. The use of sensors for moisture detection can be found, for example, in monitoring systems and networks, as a monitoring device in agriculture, and as a tool for determining soil moisture during irrigation. Furthermore, also in the field of corrosion diagnostics in the areas of infrastructure and construction. The key element in this type of sensor is the materials used and the associated availability of suitable production technologies [134].

#### 4.3.8. Electronic, Current, Energy, and Magnetic Sensors

Energy and current measurement sensors ensure the density of electrical cable isolation. Their purpose is to measure the current draw of machines. These sensors have been used in many industrial areas but have had shortcomings regarding demands in the areas of miniaturisation, energy consumption, and insufficient stability. At present, optical fibres and magnetic fluids are widely used, due to their versatility of application [135]. In the case of electronic sensors, these can be, for example, gas sensors that are capable of performing sensitive analysis in real time, thanks to their flexibility and the possibility of integration with intelligent electronics and mechanical resistance in relation to energy consumption, and respectively, the performance of electronic components. However, balancing these variables offers applications not only in the above areas, but also in aviation, aerospace, and nuclear industry [136] display devices, and there is considerable potential in the area of environmental monitoring [137]. Zhang et al. [122] described a data-driven smart production line with installed energy consumption sensors for forecast and fault diagnosis of maintenance.

#### 4.3.9. Virtual Sensors

Virtual sensors are advanced applications in the software layer of the machine that enhance the knowledge of the devices. These state-of-the-art sensors obtained data not only from physical machines (for example robots), but also the knowledge bases [138]. Al-Jlibawi et al. [107] call them adaptive soft sensors due to their low cost, parallel work, robust characteristics, easy implementation, and real-time estimation features. They used PLC and SCADA for collecting data in the refinery via a distribution control system.

#### 4.3.10. Nuclear, Chemical, Microparticles, and Nanoparticles Sensors

The last category consists of sensors that are based on modern technologies using chemical processes. These are sensors based on nanoparticles and microparticles, which enable monitoring directly inside the monitored object. Lao et al. [111] present a robust moving horizon estimation of sensor maintenance based on the observation and monitoring of chemical processes. Comparison of four simulations demonstrates the economic performance advantages of sensor-predictive maintenance. Jia et al. [139] described the design of the nanosensors for detection of antibiotics to prevent the production of resistance to antibiotics. Nanomaterial chemistry is used for developing current arsenic detection nanostructures [140]. The main advantages of nanomaterials are the high flexibility, sensitivity, compatibility, and stretchability of sensors in electronics devices [141].

## 5. Discussion

The discussion is divided into two parts. First, we discuss the sensor-based smart factory to imagine the factories of the future. Then, in the second part, we focus on insights and future research issues related to intelligent sensors.

### 5.1. Sensor-Based Smart Factory Discussion

The essence of the use of intelligent sensors based on IoT lies in smart factories, which have modern sensor technology, intelligent analytical programs, and networking components of production (machines, supplies, components, final products, equipment, etc.). Smart factories are a new way of organizing production. Their goal is to better serve customers through greater production flexibility and resource optimisation. The factories of the future combine the efficiency of mass production with custom production and optimize the supply chain in real-time thanks to the Internet connection [142]. These factories handle fluctuations in demand, which are fully automated and fault-durable. The smart factory is connected to the Internet, however it has advanced security against cyberattacks that would jeopardize production.

**Figure 10:**
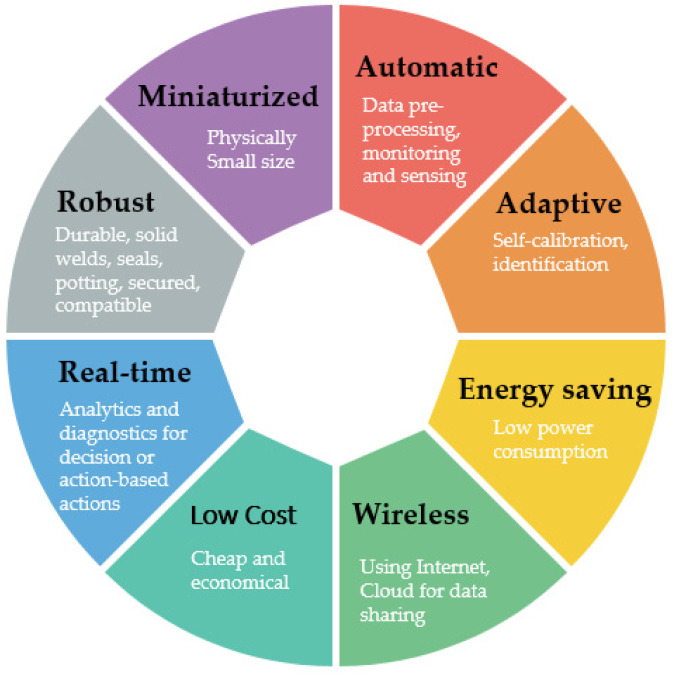
Main characteristics of intelligent sensors.

We summarised the intelligent sensor advantages in Figure 10 based on Reference [143]. Sensitivity is defined as the relation unit change between output and input. Smart sensors such as IoT devices are wireless, using the internet and usually cloud. Intelligent sensors have low power consumption, automatic diagnostics, calibration, and the ability to process and share data in real-time. Robust means good durable material, solid welds, seals, potting, chemical compatibility, secured wires, and other situational protection. Automatic diagnostics are related to the possibility for making decisions or proceeding action-based actions for control. Some authors emphasise low cost as a feature for smart sensors, but we think that it depends on user experience and sensor value added.

The transformation of a traditional factory into a smart one brings with it a higher integration of physical production with digital technologies. Sensors and actuators bring factory communication capabilities and data collection and analysis capabilities [144]. The intelligent factory brings a change from traditional automation to a fully connected and flexible system that can use a continuous flow of data from connecting operations and production systems to learn and adapt to new requirements. The production system in smart factory is different—with more resources for small-lot products, dynamic routing of production line, comprehensive connections with high-speed network infrastructure, deep convergence of physical and digital world (digital twins), self-organisation control system, and big data analytics [64]. A flexible conveying system of the production lines is designed for the main production loops (cycles), with storage loops on the production line and branch loops for customizing products. The smart factory can integrate data from corporate assets to manage production, maintenance, inventory tracking, digitize operations through the digital twin, and other technologies. In the enterprise infrastructure, smart logistics, smart grids, smart buildings, and smart distribution are interconnected. Project management is important for the successful implementation and sustainability of these systems in smart factories [145].

Due to the frequent occurrence of extraordinary situations caused mainly by external elements, there is a need to deploy more demanding control systems. Management in smart factories is decentralised. Decentralisations can offer the ability to make decisions at process locations, independent of any central entity [146]. The complexity of these environments with many simultaneous processes, most of which follow each other, as well as the enormous amount of data that sensors generate in production, can no longer be served by existing control systems based on the simple technology of recording or processing transactions. Therefore, multi-agent systems come to the light, where intelligent information agents form a network of decentralised and distributed intelligence [147,148]. Beside the existing solutions, these systems are not based on centralised control but are capable of collective self-configuration. These systems interconnect individual autonomous agents (or their digital twins) to communicate, coordinate, and cooperate to achieve a set common goal. Individual communication elements collect data as needed, which they later use to improve and optimize production.

In smart factories, thanks to intelligent sensors, each product actively participates in the production process. The components to be processed contain digital information on how to process them. They, therefore, communicate directly with robots and production machines. With the help of a chip with radio frequency identification or other sensor technology, it can control its production flow. A smart product has access to knowledge related to its structure, composition, or purpose [149]. On the other hand, thanks to this connection, the customer uses the user interface and intervenes in production in real-time. The sensors allow the customer to obtain information for creating the product specification, and its adjustment according to needs and requirements [150]. Autonomous vehicles powered by electricity are also connected to the system, ensuring the transport of stock and final products around the factory. Vehicle control is provided by a sophisticated system of sensors. Parts, materials, and components needed for production are transported to the factory when they are really needed for production (Just-in-Time system). Sensors and possibly drones constantly check stock in a smart factory [151].

**Figure 11:**
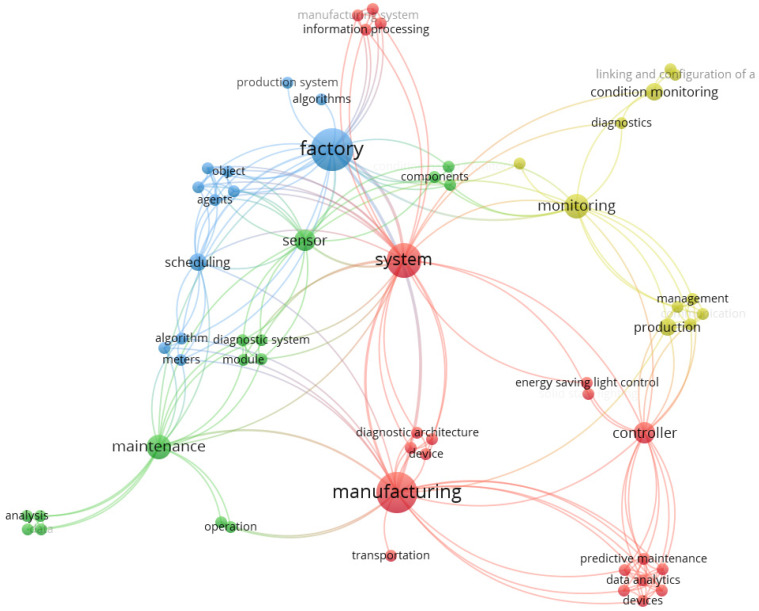
Analysis of keywords “smart” and “intelligent”.

We performed profound words’ analysis of full-text papers to find phrases containing the terms “smart” or “intelligent”. Figure 11 shows that the obtained keywords form various clusters and subclusters related to the predictive maintenance process. We identified four main components of the Smart and Intelligent Predictive Maintenance (SIPM) system for smart factories based on cluster analysis. These are the production system, the monitoring system, the factory planning system, and the maintenance system. The production system of SIPM is based on energy saving control, transportation, and economics costs, with use of controllers for predictive maintenance based on data analysis, and equipment diagnostics. The monitoring system uses condition-based diagnostics, sensors network linking management, and production—the factory planning system concerning different components of objects and agents by using algorithms and meters. The maintenance system is related to using analysis and diagnostics sensor data for predictive maintenance.

From the point of view of preventive maintenance, the machines and robots performing production communicate with each other continuously and inform each other about non-standard situations. The machines report themselves to the maintenance staff (in this sense it is a robot), besides, they precisely define the problem. The sensors in production are thus connected to other factory systems based on SCADA. All elements can minimize energy losses or use alternative energy sources for their activities [152]. Zero error rate is ensured in production using smart sensors. Smart sensors and testers monitor the quality of the final products.

We discussed the results and interpreted them in the perspective of previous studies and research. Predictive maintenances in Cavalieri-Salafia’s model [153] includes data acquisition from sensors, data manipulation (filtering, transforming, removing noise), aggregation, prediction, decision-making, scheduling, and further monitoring of status and configuration. Similarly, it describes the process of data acquisition, data processing, and machine decision-making [154]. Possible application of artificial intelligence (AI) for preventive maintenance is discussed by Carlson and Sakao [155]. Modern systems are based on the Internet of Things that enable real-time prediction and data sharing [96]. Uhlmann et al. [117] described the solution of sensor network enhanced by cloud. The edge technologies [156] allow integration between PLC and cloud for modern sensors. Miniaturisation of current sensors and nanotechnology [157] provides higher flexibility of maintenance systems. Fernandes et al. [158] emphasise the role of data visualisation, data mining, and data storage. These models have a standard data flow process which is a part of the possible prediction preparation. It is necessary to set a reliability model to analyse the dataset [159]. Ruhi and Karim [160] show that a suitable statistical model can be applied to estimate the optimum maintenance period at a minimum cost. Stodola and Stodola [161] pointed out that a useful model needs to consider human factors and related issues such as labour intensity, administrative, and human errors.

### 5.2. Discussion on Insights and Future Research Issues

We discussed practical considerations and potential avenues for future research. The following directions have been identified for potential further work.

#### 5.2.1. Wireless Network of Sensors

One of the current trends is the use of a wireless network of sensors (WSN), which according to Vlasov et al. [119] consists of intelligent sensors for sensing physical parameters. The individual sensors act as network elements (nodes) that can read, process, and transmit wireless data in smart factories. They consist of processing, communication, and the sensor unit. WSN is a network of Micro-Electro-Mechanical systems (MEMS) of sensor devices [162]. MEMS offers a high potential for condition monitoring applications. These sensors are, in comparison to industrial sensors, cost-effective, wireless, and highly integrable and configurable [117]. Part of these networks could be process controllers (actuators) that communicate data and operate some units [163]. The cyber-physical systems integrate these sensors and actuator networks into a coherent form. Vlasov et al. [119] show that the use of wireless communication channels in the monitoring system is driven by the sensor network in the shortest possible time. Uhlmann et al. [117] pointed out their main advantage—low cost. Sadiki et al. [105] recommend to check the viability of these network applications before their implementation in factories to save operation costs and improve real-time monitoring performance. Main advantages of industrial WSN, summarising Kumar et al. [163], are: minimal cost and compactness, interoperability, resistance to noise (and co-existence), self-organizing, robustness (fault-tolerance), link-reliability, energy consumption, low-delay, service differentiation and quality, scalability, predictable behaviour, multiple sources, data aggregation, and specific protocols. However, the limitations of wireless sensors are the spatial arrangement in the environment, deployment time, maintenance cost of communication channels [119], scalability, and lack of protocols. Flammini et al. [164] emphasised that fault assumption and transmission errors are more frequent in wired communication than on wired links. Thus, data quality (in terms of validity, integrity, accuracy, and reliability) is an important factor of wired networks connected to the internet [165].

#### 5.2.2. Dominance of Vibration and Temperature Sensors for Maintenance

The analysis of the types of sensors used for predictive maintenance in Section 4.3 showed that vibration and temperature sensors are most often used. According to Uhlmann [117], the number of powerful MEMS sensors such as vibration and temperature sensors has increased. Vibration can identify problems before other symptoms, such as temperature, sound, power consumption, and contaminated lubricants. When the first signs of a malfunction appear, there is usually only a few months left until a complete machine downtime. Vibration monitoring enables to determine which phase of the fault curve the machine is currently in. Excessive vibration is usually the first symptom of internal problems, such as defective bearings, imbalances, misalignments, and loose components, that shorten the life of the equipment. Only after detectable changes in vibration is it possible to detect possible errors using power, particle, or infrared sensors. Finally, errors also appear on the temperature sensors. According to Barbieri et al. [99], vibrational signals are a starting point of the component degradation model. Crucial decision lies in the selection of adequate signals for failure estimation and maintenance prediction. The further research challenge in infrared thermography is the signal and image processing to enhance the detection and simplify the interpretation of the results [166]. Akerberg et al. [167] pointed out that the requirements for vibration sensors, battery lifetime, update frequency, and security are lower than for flow, torque, or proximity sensors. The main benefits of vibration sensors are predictability of impending failures, machine safety, cost, extended maintenance intervals, machine reliability, and confidence in scheduled maintenance. In the future, these advantages resulting from the use of vibration sensors can be obtained by using new types of sensors, for example, virtual sensors or nanosensors, etc. For this reason, current research is focused on minimizing the costs and flexibility of vibration and temperature sensor solutions.

#### 5.2.3. Challenges of the Deep Learning and Big Data Analytics

Components connected to the network generate a large amount of heterogeneous data that smart industry systems must process. Large industrial data is collected from multiple sources, such as devices, products, and customers, in heterogeneous forms [108]. Their analysis is crucial for companies in almost all market sectors. Advanced analysis of big data is a way to get to know customers and devices (machine state) in detail. Companies that use it will be able to provide customers with literally “tailor-made” services, lower moods, and increase efficiency in production, including maintenance. Thanks to the current capacity of computer technology, even large volumes of data can be processed faster, which allows smart industry systems to move information in real-time. Farooq et al. [38] consider industrial prognosis and applying predictive analytics using machine learning techniques as new challenges to identify failure modes and reduce downtime. The application of the simulation enhances more possibilities for after-market services, such as maintenance [92]. Learning and intelligent techniques such as deep learning to analyse industrial big data are proposed as future directions in maintenance [103,120]. We consider the main problem in the field of data analysis to be the inability to translate and create a business case for analysts with a comprehensive view. This means that there is no concept of how to evaluate the analytical results and translate them into actions (for example, maintenance schedules). Companies often make the mistake of supplementing random findings instead of a selective and targeted approach such as predictive maintenance. Results of the analysis must lead to conclusions, which requires a properly chosen tool for data visualisation. Other possible research directions are related to virtual metrology [109].

#### 5.2.4. Challenge of Interoperability

Interconnectivity is a prerequisite for the existence of a smart industry. It is not just a peripheral connection of one device to another or a centralised connection of the machine to the control system, but a complex network. In a corporate environment, this means connecting machines, people, materials, products, information and communication technologies and systems, and, finally, data in the form of documents. Scalability enables possible network size enhancing or reduction based on the industrial requirements. This needs appropriate protocols, standards, and robust integration of hundreds of network nodes. Interoperability is linked to their compatibility, i.e., all elements of the industrial Internet of Things should be able to communicate with each other (exchange data) and interact. Through standardisation, disparate systems that work together can be soldered into a single process. Today’s factories already use sensor systems for various measurements and operations. The use of new solutions must work with these old systems. This problem is caused by a lack of robustness, time constraints, and service-differentiated protocols. One of the future solutions is, for example, the oneM2M [168] or OPC Unified Architecture [169] industry communication standards, which are platform-independent and support semantic interoperability [170]. On these platforms, data objects and each device have well-defined behaviour on the network, and the capability of horizontal communication between individual devices processing in real-time. There is a possibility to connect other components (such as plug and produce), which then have their image in the cloud.

#### 5.2.5. Challenges of Control and Maintenance Systems Decentralisation

Many of the wireless sensor networks still have centralised control (so-called “network manager”). Centralised server-client systems had one central database, which leads to data consistency, easy administration, and a high level of security. However, centralisation can result in the failure of a single point. High traffic can overload the bottleneck. A centralised “network management” system can be slow and take a vest to lose data packets. It is essential to use decentralised solutions within complex digital ecosystems, such as production factories. The responsibility and need to make operational decisions is performed at lower levels. A distributed architecture based on decentralisation is suitable for their coordination and management [171]. Coordination between the individual nodes involved in cyber-physical systems requires communication between the elements. The benefit of the distributed architecture of components connected to the network and decentralised smart industry systems is the expandability (scalability) of the network, as well as the increased resistance to failures of the network itself, individual connected systems, as well as their components. For example, Kiangala and Wang [94] proposed a decentralised monitoring system with a cloud-based dashboard displaying real-time reports for every maintenance schedule generated. Zhang et al. [122] consider as a future trend services in cyberspace that control, plan, and schedule production line items in a timely way. Smart Industry systems already use intelligent algorithms to monitor, control, manage, and plan complex processes and operations throughout the production process and supply chain. Advanced cognitive technologies will be gradually implemented in production systems to increase the autonomy of individual components of the network. These systems use the principles of collective intelligence in industrial processes, especially solutions based on multiagent systems. The future of these systems lies in achieving a high level of artificial intelligence that will use the collective knowledge of all parts of the network.

#### 5.2.6. High Potential of Virtual- and Nano-Sensors

Sensor technology, the elements informing about the state and activity of the object, is currently unprecedented and developed in various industries due to its reliability. Sensors as sources of primary information about the real environment are the input element of practically all control, measuring, and automation systems today, and together with microsystems, they accompany human activity (via biometric sensors) at almost every step. An interesting trend can be traced in the development of electronics and microelectronics in sensor systems, which include circuits for signal processing, analysis, and unification in a single compact design with a sensor element. In optimizing this process, a tighter system connection of electronics and microelectronics with related elements of optics or fine mechanics within mechatronics is increasingly being applied. The intention of such an interconnection is to achieve the development and production integration of entire systems on a single chip, from input sensors to various types of actuators. From another point of view, it is possible to see the influence of miniaturisation and new sensor types such as nanosensors. The higher resistance of the sensors to mechanical influences, acceleration, and vibrations also leads to their placement directly in the parts of the system, which in many cases gives the function of the sensor a wider range. Nanosensors have, due to miniaturisation, good preconditions for massive use in smart factories. These trends are currently aided by the search for ways to create wireless sensor networks with remote data transmission. Al-Jlibawi et al. [107] described the properties of soft (virtual) sensors and their function in parallel work with physical sensors and industrial processes. Virtual sensors are used for damage detection [172], industrial robot interaction [173], identification, and approximation tasks, or for digital twin applications. On-line evaluation of input effects directly in the actuator can then lead to a “sensor-less” system, whose other advantage, in addition to lower one-time investment costs, should be the greater reliability of the system with protection against possible outages. Li et al. [165] described automatic control systems without sensors based on wireless nodes, RIFID, and programmable logic controller (PLC). Automated devices perform their tasks based on industrial wireless nodes fully responsible for communication. Controllers have the automation control function based on the received commands from other devices in the network.

#### 5.2.7. Challenges of Availability and Reconfigurability of Sensors

The development of industry systems is progressing towards their maximum modularity and capability of autonomous reconfiguration based on automatic situation recognition. The self-organisation of production processes therefore also includes reconfigurability, characterised as independent adaptability to internal and external conditions. Condition-based systems are being developed in the field of maintenance. The main problem in the future may be the use of old sensors (installed on older devices in production), which will lead to unacceptably high manufacturing and maintenance costs in the long term. Kozlowski et al. [110] mentioned the unavailability of devices (CNC machines) on the market to structurally optimally distributed sensors on machine parts. Related to this issue is the problem of today’s industrial practice—the lack of appropriate measurement signals. The result is the need for additional installation of sensors on the device. A problem with the weak signal from the sensors can lead to injury, product losses, and production outages. Even small and temporary communication errors can cause significant production interruptions [167]. This production issue can be caused by signal inference from devices that operate on the same frequency. It means that at the same time, waves and signals coexist in a given environment, which disrupt the function of the sensors. This problem can be described as a real-time availability issue. Lao et al. [111] show that preventive maintenance of sensors and actuators in real-time can significantly mitigate the damage from production losses, process upsets, and downtime based on specific routine regulations.

#### 5.2.8. Security and Safety Challenge

The severity of cyber security issues is growing, along with our growing dependence on technology, both personally and socially. In production, important data is collected by increasingly connecting production systems or even the robots themselves to computer networks, sometimes with communication to the cloud, and new security risks arise. Basic cyber security principles can significantly improve the security of connected devices. The contemporary challenge of safety is the security of the stored data from sensors. In addition to the sensors that collect data, which is a physical prerequisite for the Industry 4.0 concept, the sovereignty of the data is important [174], especially from a psychological point of view. Only enterprises that trust the security of their networks are also willing to store and share their data. Sensor security does not require high demands on battery capacity. However, security is considered too complicated, especially for IoT, due to the use of various techniques such as keying to protect authentication, cryptographic code verification, security gateways [175], safety protocols, remote wireless security management [176], or security analysis to detect attacks. Almost all interconnected systems have their weaknesses that their creators are unaware of. For this reason, prevention is particularly important [167]. An important factor due to the number of interconnected devices is the cost of security. Gungor and Hancke [177] point out that security is a crucial feature in designing wireless sensor networks. Safe communication protects against external attacks and intrusion.

## 6. Conclusions

The fourth industrial revolution is permeating the industry, enabling an increasing number of enterprises to have an incomparably greater overview of their production and maintenance activities than ever before. The deployment of highly reliable and low-maintenance devices contributes to the precise planning of production capacity and equipment’s associated maintenance.

The first research question relates to the contemporary trends in the maintenance processes of smart factories. The number of papers discussing the key terms sensors, smart factories, and preventive maintenance increased over time, mostly in the last years. We found that the contemporary burst trend is related to Industry 4.0 technology. Predictive maintenance, smart factories, and intelligent sensors publications, together concerned topics mainly related to deep machine learning, Internet of Things, and big data analytics. The maintenance process in smart factories is based on digitisation, data-driven manufacturing, agent-based systems, and digital twins. Intelligent sensors in such factories use edge, fog, and deep learning methods for control of manufacturing processes. In the future, Internet and blockchain will be important for predictive maintenance.

Smart and intelligent predictive maintenance is characterised to answer a second research question. Here, the results show four different types of maintenance used in smart factories—Industry 4.0 for predictive maintenance, smart manufacturing for condition-based maintenance, fault diagnosis for maintenance and prognostics, and remaining useful life analysis. The importance of predictive maintenance is also growing due to the growing number of robots, digitisation, and artificial intelligence introduced into production lines to automate routine activities.

Following the third question’s answer, we can state that the three types of sensors are mainly used for predictive maintenance in smart factories. Firstly, intelligent sensors which have the potential to connect to higher-level systems. Furthermore, there is a possibility to connect these intelligent sensors to the internet—to build up the IoT devices. Finally, we can use the gathered data in cloud-related technologies. The most prevalent methods used for collecting and monitoring machines and devices are vibration analysis [120], SCADA systems, CNC machine sensors, and PLCs. Based on the deep analysis, we conclude that the current trend, insights, and future research issues are characterised by:

- Usage of multisource wireless networks of sensors in predictive maintenance.
- Dominance of vibration and temperature sensors for predictive maintenance.
- Challenges of the big data analytics and deep learning techniques.
- Challenges of interoperability of multiple sensors and maintenance systems.
- Decentralisation of maintenance control systems.
- High potential of virtual sensors and nanosensors for the future.
- Challenge of availability and reconfigurability of sensors.
- Security and safety of sensor data.

Based on the results synthesis, we proposed the Smart and Intelligent Predictive Maintenance (SIPM) system for smart factory concerning four major subsystems: production, monitoring, planning, and maintenance. These subsystems communicate and collaborate through modern IoT and cloud-based technologies. Their main advantage is real-time management and planning to reduce the economic costs caused by production downtime.

From a managerial point of view, the predictive maintenance system in smart factories is an early warning, especially in high-risk industries. The ability to detect weak signals of potentially significant strategic impact is a welcome positive in a turbulent business environment. The system of predictive maintenance does not reduce the responsibility or the possibility of personal development of employees, but it must be stimulated by responsible managers. It offers the possibility to reduce the number of hierarchical levels between managers and ordinary employees, so that you can bring about higher employee autonomy and help other innovation processes to be implemented effectively. The challenge for managers today is to select criteria based on which they will be able to select intelligent sensors for smart factories. There is a wide range of sensors on the market and the authors most often state the criteria of sensor sensitivity, sensor cost, flexibility, and size (miniaturity).

Further research may comprise the advanced machine learning methods as deep learning, data-driven algorithms. The new concept called “Machine as a Service” (MaaS) takes over the software as a service (SaaS) model and is another trend suitable for future research. An interesting direction of future research concerns building performance model evaluation related to the reasonable cost. The cost/benefit analysis of using preventive tools in contrast to sustainability requirements is challenging for research.

This work suffers from several limitations, notably related to publication collections, filtering, and analysis. The search strategy is biased by the problematic synonym of the term “factory”. Primarily, the term “plant” is not interchangeable in the same meaning. The study is limited because we omitted highly cited publications related to medicine in burst analysis. Our goal was to bring the reader closer manufacturing- and production-related publications. Investigated trends in burst detection analysis have weights based on occurrence in publication titles. Results do not show the quality of publication based on the times cited. Thus, we instead present examples of highly cited publications of most important burst terms. The lack of a comprehensive review due to a steadily increasing number of related works is another notable limitation of this study.

## Figures and Tables

**Table 1:** PICO (Problem/context, Intervention, Comparison, Outcomes) logic grid.

	Context (How?)	Interventions (Which?)	Outcomes (What?)
Topic	Smart Factory	Intelligent sensors	Predictive Maintenance
Synonyms	Intelligent Factory	Smart sensors	Smart maintenance
	Smart manufacturing		
	Smart production		Intelligent maintenance
Keywords	System	Internet of things	Optimisation
	Intelligent control	Internet	Management
	Automation	Wireless networks	System
	Neural networks	Security	Reliability
	Simulation	Network	Diagnosis
	Multi-agent systems	Big Data	Condition monitoring
	Robotics	Machine Learning	Prognostics
	Scheduling	Management	Machine learning
	Sensors	Cloud Computing	Big Data
	Artificial intelligence	Energy	Industry 4.0
	Pattern recognition	Smart City (home, grid)	Preventive maintenance
	Fuzzy control	Cloud	Performance
	Internet	Monitoring	Prediction
	Optimisation	Privacy	Classification
	Software	Activity recognition	Risk
	Expert systems	Classification	Fault diagnosis
	Learning	Cyber-Physical System	Identification
	Mechatronics	Blockchain	Internet of Things
	Data mining	Deep Learning	Facility management

**Table 2:** Search strategy for burst detection analysis (1 December 2020).

Searches	Terms/Thesaurus
1. Smart factory/production	(“factory” OR “factories” OR “production” OR “manufacture*”) AND (“smart” OR “intelligent”)
2. Intelligent sensors	(“sensor” OR “sensors”) AND (“smart” OR “intelligent”)
3. Predictive maintenance	(“maintenance”) AND (“smart” OR “intelligent” OR “predictive”)
Query	TOPIC (1), TOPIC (2), TOPIC (3)

**Table 3:** Results of topics searched for burst detection analysis (1 December 2020).

Topic	Smart Factory/Production	Intelligent/Smart Sensors	Predictive/Smart Maintenance
Web of Science	26,764	53,368	13,994
Scopus	65,350	103,480	28,803

**Table 4:** Search strategy in the second phase (1 December 2020).

Searches	Terms/Thesaurus
1. Smart factory/production	(“factory” OR “factories” OR “production” OR “manufacture*”)
2. Intelligent sensors	(“sensor” OR “sensors”)
3. Predictive maintenance	(“maintenance”)
4. Smart/intelligent	(“smart” OR “intelligent”)
Query	1 AND 2 AND 3 AND 4

**Table 5:** The number of papers from Web of Science (WoS) and Scopus bibliographic databases.

	Article	Other	Total
WoS	102	148	250
Scopus	186	454	640

**Table 6:** Maintenance process characteristics *.

Authors	Maintenance Characteristics	Type
Al-Jlibawi et al. [107]	The estimation of octane number in the gasoline formed by refineries is presented. This type of virtual instrument was designed with the aim of replacing measuring hardware during maintenance tasks. The results of proposed soft sensor mathematical model will compare with laboratory results for product quality purposes and the errors between predictive results and actual results will be feedback to improve the control system performance in particular and manufacturing systems in general.	I4
Barbieri et al. [99]	Manufacturers can take advantage of this methodology to integrate autonomous maintenance policies as features in their machines, keeping their expertise with standard automation platforms.	RUL
Bekar et al. [108]	The analysis resulted in dimension reduction of feature space and also clustering of data points for understanding of the outliers in anomaly clusters by incorporating maintenance domain knowledge. These knowledge discovery methods using unsupervised ML should be the first step within predictive maintenance implementations.	I4
Farooq et al. [38]	We established a genetic algorithm (GA) based on multi-sensor performance assessment and prediction procedure for the spinning system. We have successfully adopted a GA-based prediction process for our spinning system, which worked as an intelligent maintenance and scheduling system for health assessment.	CbM
Goodall et al. [92]	The adaptive remanufacturing simulation is based upon a generic view of material flow in remanufacturing operations where a core can be in one of two states: waiting or processing.	I4
Chien and Chen [109]	This study developed an effective approach that integrated partial least square and exponentially weighted moving averages approaches for tool health status monitoring and prediction to effectively derive the optimal maintenance strategies via transforming and analysing big data collected from the sensors, including the SVID data and FDC (fault detection and classification) parameters.	RUL
Kiangala and Wang [94]	An effective predictive maintenance strategy for a conveyor motor based on Industry 4.0 concepts was proposed. In the proposed strategy, we have rigorously analysed real-time vibration speed data collected from a vibration sensor mounted on the conveyor motor and connected to a Siemens S7-1200 PLC.	SFD
Kozlowski et al. [110]	The proposed method has been verified using data from a CNC machine monitoring system. The objective of RUL (Remaining Useful Life) classification and prediction was to prevent the manufacturing of details that fall short of quality requirements when the process is performed with the use of a blunt tool.	RUL
Kumar et al. [102]	In the context of condition-based maintenance, the proposed framework allows us to overcome the tedious and often impossible task of “labelling” dataset health-states, and hence, improves autonomy of techniques for diagnostics.	RUL
Lao et al. [111]	In this work, handling scheduled preventive sensor maintenance via the Lyapunov-based economic model predictive control(EMPC) system design is considered. A robust moving horizon estimation (RMHE) scheme is developed that accommodates a varying number of sensors to continuously supply accurate state estimates to an EMPC (economic model predictive control) system.	SFD
Li et al. [96]	The proposed DAMSID (Deep Adaboost Machine Learning assisted Sampling Design) offers a team training technique based on classifiers offline to tackle by CBM with floats of ideas and information on irregularities, which represents the specific segments (linear four rates and classifier of nominal and continuous) to be strengthened by modifying the following conditions.	CbM
Lin et al. [103]	This work proposes an ensemble learning algorithm using DAMSID that supports the use of classifiers to cope with three-stage CBM with concept drifts and imbalance data.	CbM
Musselman and Djurdjanovic [104]	Experiments were run to establish the tension estimation variance when a human completely executed the manual technique (standard approach) and the tension estimation variance when the newly designed contact-based device was used for belt excitation and signal collection.	CbM
Park et al. [112]	In this study, the lifespan of the servo motor was estimated through accelerated degradation testing methods based on a new system degradation assessment method, which estimates the fault of the system using observer-based residuals with encoder data obtained from internal instrumentation, and the importance of the maintenance for machineries within manufacturing sites.	SFD
Peng et al. [113]	As a result, in the pursuit of the so-called smart factory and the enhancement of the production process, as well as attenuation of numerous human maintenance efforts, a graphical histogram algorithm (GHA) health condition diagnosis and monitoring strategy is proposed.	SFD
Peng and Tsan [98]	With these applications, unscheduled shut down for inspection can be avoided, and preventive maintenance can be deployed when the online sensor is identified as faulty.	SFD
Sadiki et al. [105]	We evaluated our developed wireless sensor network application in the context of maintenance monitoring on realistic networks using the Instant Contiki operational system environment. We used Cooja simulator to investigate the robustness of our system in a scenario where nodes (sensors) will collect data on a real-time basis and transmit to the central node.	CbM
Shan et al. [114]	Preventive maintenance of intelligent manufacturing equipment is carried out to reduce the failure rate of intelligent manufacturing equipment and promote the development of the new generation of intelligent manufacturing systems.	I4
Tarashioon et al. [115]	System checks if it is capable of doing self-maintenance, otherwise it will request maintenance from operators (human maintenance instead of system self-maintenance).	RUL
Tsao et al. [116]	This study incorporates Industry 4.0, which considers predictive maintenance, into the imperfect production systems into economic production quantity (EPQ) models. The predictive maintenances could be implemented by using sensors and data analysis, which maintain production systems before they become ‘out of control’.	SFD
Uhlmann et al. [117]	This presented solution can be used to monitor production systems and their wear-susceptible and critical components such as ball screw and bearings. This solution is to realise, due to decentral data processing on the sensor nodes, the concentration of data and services in the cloud. Mobile provision of data and merging varied distributed sensors into a sensor network.	I4
Villalobos et al. [118]	Alarms can allow the operators in the plant to conduct proactive management of the different controls in the machine for predictive maintenance of the equipment.	CbM
Vlasov et al. [119]	Model for optimising predictive maintenance of equipment using wireless sensor networks based on minimising the costs of maintenance, diagnostics, and deployment of the equipment.	SFD
	Monitoring system is proposed. The presented concept of a system of predictive maintenance based on sensor networks allows real-time analysis of the state of equipment.	
Yan et al. [120]	The findings of this paper indicated that multisource heterogeneous data can provide new solutions for predictive maintenance, scheduling, and machining process optimisation for energy saving.	RUL
Zhang et al. [121]	As a key component of mechanical systems, rotatory machine has significant influence upon the whole system, and the degradation of rotatory machine may lead to deadly industrial accidents. Therefore, prognostics and health management (PHM) technology is highly desired to reduce maintenance costs and improve system reliability and safety.	RUL
Zhang et al. [122]	Based on the prediction of energy consumption, it is possible to provide proactive maintenance on equipment with malfunction and potential failure.	I4

* Acronyms: I4 (Industry 4.0 for predictive maintenance in general), CbM (Smart manufacturing for condition-based maintenance), SFD (Condition, state, and fault diagnosis for maintenance, RUL (Prognostics and health management for remaining useful life).

**Table 7:** Classification of Industry 4.0 technologies based on obtained keywords’ co-occurrences.

**A: Intelligent Sensors**	
Sensor/Actuator	Kumar et al. [102], Lao et al. [111], Park et al. [112], Peng et al. [113], Peng and Tsan [98], Tarashioon et al. [115], Tsao et al. [116]
Automation	Musselman and Djurdjanovic [104]
**B: Cloud-related Technologies**	
Cloud	Kiangala and Wang [94], Uhlmann et al. [117], Vlasov et al. [119]
Cloud/edge computing	Barbieri et al. [99], Yan et al. [120], Zhang et al. [122]
Big Data	Barbieri et al. [99], Kozlowski et al. [110], Chien and Chen [109], Villalobos et al. [118], Yan et al. [120], Zhang et al. [122]
RFID	Goodall et al. [92], Sadiki et al. [105], Vlasov et al. [119], Zhang et al. [122]
PLC	Al-Jlibawi et al. [107], Barbieri et al. [99], Kiangala and Wang [94]
**C: Internet of Things Technologies**	
Internet of Things	Farooq et al. [38], Li et al. [96], Lin et al. [103], Sadiki et al. [105], Shan et al. [114], Uhlmann et al. [117], Vlasov et al. [119]
CPS system	Farooq et al. [38]
SCADA	Farooq et al. [38], Al-Jlibawi et al. [107], Kiangala and Wang [94]
Machine learning	Zhang et al. [121]
Artificial Intelligence	Bekar et al. [108]

**Table 8:** Methods and sensors’ data.

Authors	Method	Data Source
Al-Jlibawi et al. [107]	simulation	DCS (distributed control systems), PLC, or SCADA in refinery
Barbieri et al. [99]	case study	Alternating current (AC) motor (machinery), Pronistia dataset
Bekar et al. [108]	case study	Machine motor
Farooq et al. [38]	case study	SCADA in spinning factory, spinning frame JWF1562
Goodall et al. [92]	simulation	RFID in remanufacturing facility
Chien and Chen [109]	case study	health status of plasma enhanced chemical vapor deposition (PECVD) chamber tool in TFT(thin film transistor) and LCD (liquid crystal display) company
Kiangala and Wang [94]	experiment	SCADA, conveyor motors
Kozlowski et al. [110]	case study	CNC cutter machine sensors for milling of thin-walled aircraft engine components
Kumar et al. [102]	case study	CNC machine sensors
Lao et al. [111]	simulation	chemical product concentration and temperature profiles
Li et al. [96]	experiment	test data from IoT devices and detectors
Lin et al. [103]	experiment	test data from IoT in smart factory
Musselman and Djurdjanovic [104]	experiment	automated storage/retrieval systems (belt-driven material handling device) in semiconductor industry
Park et al. [112]	experiment	servo motor testing data in smart factory
Peng et al. [113]	experiment	NI-PXI (PCI extensions for instrumentation) and NI-Compact data acquisition from production lines in China Steel Corporation
Peng and Tsan [98]	experiment	production line machines
Sadiki et al. [105]	case study	industrial machine behaviour
Shan et al. [114]	simulation	welding robot in automotive production line
Tarashioon et al. [115]	experiment	LED (light-emitting diode) lighting system technologies
Tsao et al. [116]	simulation	production system and production lines
Uhlmann et al. [117]	experiment	ball and screw monitoring of machine tools
Villalobos et al. [118]	case study	melting and extruder machines in plastic bottles production plant (Capital Equipment Manufacturer)
Vlasov et al. [119]	case study	the supporting bearing of electric machines (AC motors)
Yan et al. [120]	case study	vibration signal from the cutter (CNC machine), images captured by a 3D laser scanner, acoustical signal collected by sound sensors, and power data obtained from power meters
Zhang et al. [121]	experiment	vibration data were collected by the bearing testbed (rotatory machine)
Zhang et al. [122]	case study	LED epoxy moulding compound production line

**Table 9:** Sensors’ characteristics.

Authors	Sensor Type	Sensor Description
Al-Jlibawi et al. [107]	virtual	software sensor
Barbieri et al. [99]	vibration	B&R X20CM4800X, Beckhoff EL3632
Bekar et al. [108]	temperature, vibration	not available
Farooq et al. [38]	speed, vibration	not available
Goodall et al. [92]	position tracking	RFID for traceability
Chien and Chen [109]	multiple (temperature, pressure, flow, position, power)	Silane (SiH4) flow sensor, radio frequency plasma generation sensor, peak-to-peak voltage radio frequency sensors
Kiangala and Wang [94]	vibration	SiemensS7-1200 PLC, vibration sensor (4–20 mA analogue input, www.ifm.com/gb/octavis, accessed on 19 February 2021.)
Kozlowski et al. [110]	torque	three-axis sensor for torque signals, chuck-mounted sensor
Kumar et al. [102]	torque, force	Kistler 9257B piezodynamometer (sampled at 250 Hz)
Lao et al. [111]	multiple (flow, temperature, volatility, energy, volume, gas, chemical)	sensors and actuators in chemical process
Li et al. [96]	not available	not available
Lin et al. [103]	not available	not available
Musselman and Djurdjanovic [104]	multiple (location, acoustic, tension)	not available
Park et al. [112]	multiple (torque, temperature, position)	not available
Peng et al. [113]	accelerometer	Integrated Electronics Piezo-Electric (IEPE) sensor
Peng and Tsan [98]	accelerometer	IEPE sensor
Sadiki et al. [105]	multiple (temperature, vibration)	Tmote sky (wireless sensors module) and Z1 mote (ADXL345 accelerometer and TMP102 temperature sensor)
Shan et al. [114]	movement	not available
Tarashioon et al. [115]	light, temperature	LED sensors architecture
Tsao et al. [116]	not available	not available
Uhlmann et al. [117]	vibration, temperature	Micro-Electro-Mechanical System (MEMS) of vibration sensors (LIS3DH)
Villalobos et al. [118]	temperature, pressure, speed	not available
Vlasov et al. [119]	vibration	vibration sensors network
Yan et al. [120]	multiple (vibration, optical, acoustical, power)	3D scanner (Microscope OLS3000), power meters type CW240
Zhang et al. [121]	vibration	bearing testbed
Zhang et al. [122]	energy	smart meter (Schneider PM5350)

## Data Availability

Not applicable.

## Supplementary Materials

The PRISMA Checklist (Table S1) and burst analysis results (Figures S1–S3) are available online at https://www.mdpi.com/1424-8220/21/4/1470/s1, Figure S1: The burst detection in topic Smart Factory/production based on WoS data, Figure S2: The burst detection in topic Smart Sensors based on WoS data, Figure S3: The burst detection in topic Smart/predictive maintenance based on WoS data, Table S1: The PRISMA Checklist.
